# ITCH-dependent proteasomal degradation of c-FLIP induced by the anti-HER3 antibody 9F7-F11 promotes DR5/caspase 8-mediated apoptosis of tumor cells

**DOI:** 10.1186/s12964-019-0413-8

**Published:** 2019-08-23

**Authors:** Christophe Le Clorennec, Yassamine Lazrek, Olivier Dubreuil, Carla Sampaio, Christel Larbouret, Romain Lanotte, Marie-Alix Poul, Jean-Marc Barret, Jean-François Prost, André Pèlegrin, Thierry Chardès

**Affiliations:** 1 Institut de Recherche en Cancérologie de Montpellier (IRCM), INSERM U1194, Université de Montpellier, Institut régional du Cancer de Montpellier (ICM), F-34298 Montpellier, France; 2 GamaMabs Pharma SA, Centre Pierre Potier, F-31106 Toulouse, France; 3 Laboratoire d’Immunologie et d’Immunothérapie des Cancers, EA7269, Université Bourgogne Franche-Comté, F-21000 Dijon, France; 4 Centre National de la Recherche Scientifique (CNRS), Paris, France; 5 Present Address: UCSD School of Medicine, Moores Cancer Center, La Jolla, CA 92093-0815 USA; 6 Present Address: Institut Pasteur de Guyane, F- 97306 Cayenne, France

**Keywords:** Cancer, HER3, Antibody, Apoptosis, C-FLIP, ITCH

## Abstract

**Background:**

HER3/ErbB3 receptor deletion or blockade leads to tumor cell apoptosis, whereas its overexpression confers anti-cancer drug resistance through upregulation of protective mechanisms against apoptosis. We produced the anti-HER3 antibody 9F7-F11 that promotes HER3 ubiquitination and degradation via JNK1/2-dependent activation of the E3 ubiquitin ligase ITCH, and that induces apoptosis of cancer cells. Cellular FLICE-like inhibitory protein (c-FLIP) is a key regulator of apoptotic pathways. Here, we wanted to determine the mechanisms underlying the pro-apoptotic effect of 9F7-F11.

**Methods:**

Anti-HER3 antibody-induced apoptosis was assessed by western blot, and by flow cytometry measurement of Annexin V/7-AAD-labelled tumor cells (BxPC3, MDA-MB-468 and DU145 cell lines). c-FLIP/ITCH interaction and subsequent degradation/ubiquitination were investigated by co-immunoprecipitation of *ITCH*-silenced vs scramble control cells. The relationship between ITCH-mediated c-FLIP degradation and antibody-induced apoptosis was examined by western blot and flow cytometry of tumor cells, after *ITCH* RNA interference or by pre-treatment with ITCH chemical inhibitor chlorimipramine (CI).

**Results:**

Following incubation with 9F7-F11, cancer cell apoptosis occurs through activation of caspase-8, − 9 and − 3 and the subsequent cleavage of poly (ADP-ribose) polymerase (PARP). Moreover we showed that ubiquitination and proteasomal degradation of the anti-apoptotic protein c-FLIP was mediated by USP8-regulated ITCH recruitment. This effect was abrogated by *ITCH*- and *USP8*-specific RNA interference (siRNA), or by the ITCH chemical inhibitor CI. Specifically, *ITCH* silencing or CI blocked 9F7-F11-induced caspase-8-mediated apoptosis of tumor cells, and restored c-FLIP expression. *ITCH*-silencing or CI concomitantly abrogated HER3-specific antibody-induced apoptosis of Annexin V/7-AAD-labelled BxPC3 cells. 9F7-F11 favored the extrinsic apoptosis pathway by inducing TRAIL-R2/DR5 upregulation and TRAIL expression that promoted the formation of death-inducing signaling complex (DISC), leading to caspase-8-mediated apoptosis. Incubation with 9F7-F11 also induced BID cleavage, BAX upregulation and BIM expression, which initiated the caspase-9/3-mediated mitochondrial death pathway. The anti-HER3 antibody pro-apoptotic effect occurred concomitantly with downregulation of the pro-survival proteins c-IAP2 and XIAP.

**Conclusions:**

The allosteric non-neuregulin competing modulator 9F7-F11, sensitizes tumor cells to DR5/caspase-8-mediated apoptosis through ITCH-dependent downregulation of c-FLIP.

**Graphical abstract:**

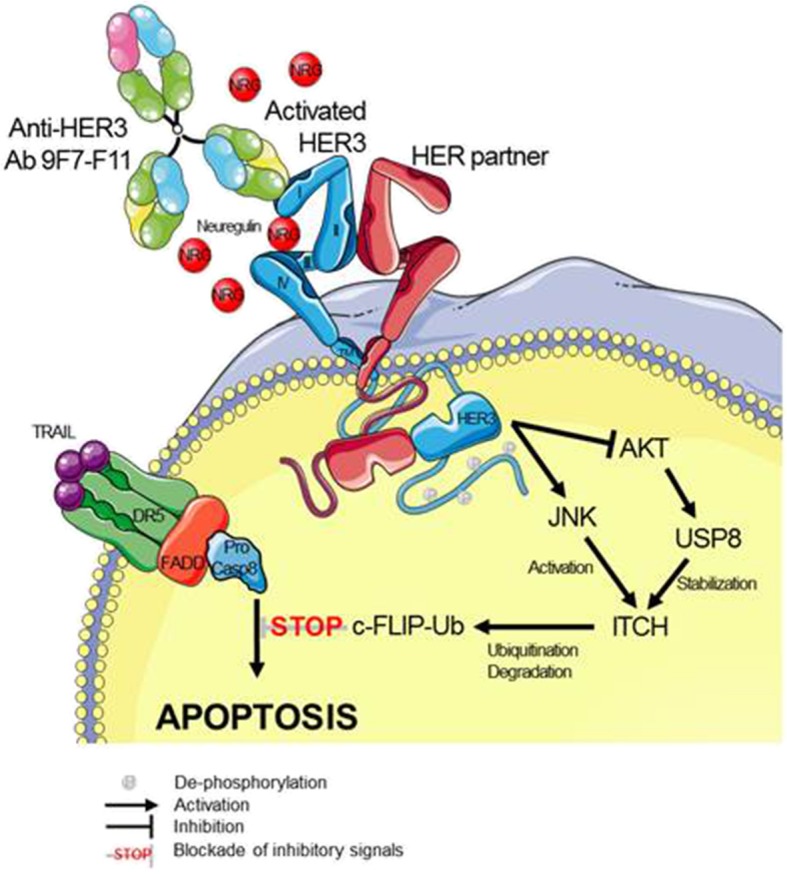

**Electronic supplementary material:**

The online version of this article (10.1186/s12964-019-0413-8) contains supplementary material, which is available to authorized users.

## Background

Apoptosis is a cell death type that is essential during embryonic development and for homeostasis maintenance. It is also one of the processes deregulated during cell transformation. In many cancers, activation of the HER3 (ErbB3) receptor frequently results in strong and aberrant activation of the AKT pathway, a critical oncogenic stimulus that leads to apoptosis resistance. HER3 knockdown [1] or pharmacological blockade [2] directly restores tumor cell-specific apoptosis. Conversely, HER3 overexpression confers drug resistance to paclitaxel in breast cancer via upregulation of survivin, a pro-survival protein that inhibits apoptosis [3]. In this case, targeting HER3 with antibodies indirectly counteracts drug resistance by favoring tumor cell apoptosis [4].

Apoptosis is induced through two main pathways. The extrinsic pathway is activated by the binding of specific ligands (e.g., FASL, TRAIL, and TNF) to their death receptors (DR) at the cell surface (FAS/CD95, DR4/TRAIL-R1 and DR5/TRAIL-R2, TNFR1 and TNFR2) [5–7]. These ligand/receptor complexes recruit adaptor molecules, such as Fas-associated protein with death domain (FADD), on their death effector domains (DED), leading to the formation of Death-Inducing Signaling Complexes (DISC) that in turn recruit pro-caspase 8. Following autocatalytic cleavage [8, 9], activated caspase-8 induces cleavage and activation of caspase-3, − 6 and − 7 directly in type I cells [10], or indirectly in type II cells, by BID cleavage into the truncated form tBID [11] that activates the intrinsic apoptotic machinery by formation of tBID/BAX complexes in mitochondria [12]. These complexes form mitochondrial pores for cytochrome C release that promotes apoptosome formation and then caspase-9 cleavage/activation to induce apoptosis [13]. DISC-induced apoptosis is negatively regulated by a short-lived protein called FLICE-like inhibitory protein (c-FLIP) [14], a competitive mimetic of pro-caspase 8 [15–17] with two DEDs, like the initiator caspase. However, due to the absence of the catalytic cysteine residue present in pro-caspase 8, c-FLIP is inactive, and thus is considered to be an anti-apoptotic protein. c-FLIP also blocks the intrinsic apoptotic pathway by competing with caspase-8 to prevent BID activation. c-FLIP is upregulated in many cancers (e.g., pancreatic [18], breast [19], prostatic [20], and colorectal [21] cancer, glioblastoma [22], Burkitt and non-Hodgkin lymphoma [23, 24]). c-FLIP upregulation promotes defects of DR-mediated apoptosis and resistance to several anti-cancer drugs [25]. However, c-FLIP is an unstable protein with rapid turnover via ubiquitination and proteasomal degradation by specific E3 ubiquitin ligases, such as ITCH [26–28]. Cisplatin treatment sensitizes tumor cells to apoptosis by favoring ITCH-mediated c-FLIP downregulation [29, 30]. Conversely, cystatin B inhibits TRAIL-induced apoptosis in melanoma cells by protecting c-FLIP from degradation by ITCH [31].

We previously showed that several anti-HER3 antibodies, including the allosteric non-neuregulin competing modulator 9F7-F11 [32], induce ITCH-mediated HER3 degradation [33] and promote apoptosis in tumor cells [2]. Among numerous anti-HER3 antibodies described [34, 35], 9F7-F11 is the only anti-HER3 antibody whose binding and affinity to HER3 are enhanced in the presence of neuregulin 1 (NRG1) [32]. This property translated in vivo into an efficient anti-tumor activity in NRG1-addicted and NRG1-rearranged cancer cells [32], making it a first-in-class antibody. Here, we asked whether its pro-apoptotic effect was regulated by ITCH-dependent c-FLIP expression regulation. We found that 9F7-F11 sensitizes tumor cells to DR5/caspase-8-mediated apoptosis through ITCH-dependent downregulation of c-FLIP.

## Methods

### Cell lines

The BxPC3 (pancreas), MDA-MB-468 (breast), and DU145 (prostate) human cancer cell lines were obtained from the American Type Culture collection (ATCC) (Rockville, MD), and grown as recommended by ATCC. All cell lines were free of mycoplasma contamination (determined by the MycoAlert™ Detection Kit (Lonza, Switzerland) and were authenticated by short tandem repeat profiling using the Promega PowerPlex 21 System. These three cell lines both expressed EGFR, HER2 and HER3 receptors, and the HER3 ligand neuregulin, and are all equipped with the ITCH/USP8/USP9X machinery (Additional file 1: Figure S1). We previously demonstrated that the anti-HER3 antibody 9F7-F11 induced HER3 ubiquitination and degradation in these three cell lines through a JNK-dependent ITCH activation [33]. Furthermore, 9F7-F11 reduced HER2/HER3 heterodimerization, increased HER3 homodimers, without affecting HER2 homodimers in HER2/HER3-transfected 3T3 cells [36].

### Antibodies and other reagents

The anti-HER3 antibody 9F7-F11 has been described elsewhere [2, 32, 33, 36]. Rabbit monoclonal antibodies against HER3 and phosphorylated (p) HER3 (Tyr1289), PARP (clone 46D11), cleaved caspase-8 (Asp391; clone 18C8), caspase-9 and cleaved caspase-9, caspase-3 (clone 8G10) and cleaved caspase-3, XIAP, DR5 (clone D4E7), BIM, c-FLIP (clone D16A8), USP9X, β-actin and β-tubulin were from Cell Signaling Technology (Danvers, MA). The mouse monoclonal antibody against ITCH was from BD Biosciences (San José, CA; ref.611199). The rabbit polyclonal antibodies against HER3 (clone C17), USP8, USP9X, c-FLIP_L/S_ (clone H-202), BAX, FAS, TRAIL, DcR2, cIAP2, and BID were from Santa Cruz Biotechnology (Santa Cruz, CA). For detection of activated ITCH, a rabbit anti-pITCH (Thr222) antibody from Millipore (Billerica, MA) was used. The human recombinant NRG1-β1 extracellular domain (ECD) was from RD Systems (Minneapolis, MN) and was used at 100 ng/ml. The proteasome inhibitor MG132 and chlorimipramine (CI) were from Sigma-Aldrich (St Louis, MO). The control IgG antibody used for co-immunoprecipitation experiments was from Santa Cruz Biotechnology.

### siRNA transfections

2 × 10^6^ BxPC3 cells were plated in 10 cm-dishes with RPMI medium without antibiotics until 50% confluence. Cells were then transfected with 50 nM of pools of four different siRNAs against human *ITCH* or *USP8*, or scramble control (ON-TARGETplus SMART pool, Dharmacon, Germany) in OptiMEM medium using Interferin™ (Life Technologies, Carlsbad, CA). After 4 h of transfection, medium was replaced by RPMI for another 48 h before using the cells for apoptosis measurement by flow cytometry, or for another 72 h before using the cells for western blot analysis.

### Annexin V/7-AAD apoptosis measurement by flow cytometry

Cell apoptosis was assessed using Annexin V/7-Amino actinomycin D (7-AAD) apoptosis kit (Beckman Coulter-Immunotech, Marseille, France) according to the manufacturer’s instructions. Briefly, 10^5^ serum-starved BxPC3 cells were transfected with 50 nM *ITCH* or scramble control siRNAs, as described above. Alternatively, BxPC3 cells were pre-treated with 15 μM of ITCH chemical inhibitor CI. After 48 h, cells were washed and treated with 50 μg/ml of anti-HER3 antibody 9F7-F11, with or without 100 ng/ml of NRG1 for 96 h. As positive control, 300 nM staurosporine (Sigma, Saint-Louis, MO) was incubated with BxPC3 cells for 6-20 h. After Annexin V/7-ADD labeling of treated cells, data were acquired on a Gallios flow cytometer and analyzed with the Kaluza software (Beckman Coulter). All experiments were performed in triplicates.

### Cell lysis and immunoprecipitation

10 × 10^6^ BxPC3 cells were lysed in CHAPS buffer (Sigma-Aldrich) containing the protease inhibitor cocktail V (Calbiochem, Billerica, MA) and the phosphatase inhibitor cocktail II (Sigma-Aldrich). For c-FLIP_L/S_ immunoprecipitation (Fig. 4), 2 mg of each total cell lysate was pre-cleared by overnight addition of 50 μl of magnetic beads (Dynabeads™; Life Technologies), to capture and remove the anti-HER3 antibody 9F7-F11. Supernatants (2 mg) were then incubated with 2 μg of the anti-c-FLIP_L/S_ antibody H-202, which recognizes both c-FLIP_L_ and c-FLIP_S_, at 4 °C for 6 h before overnight incubation with 20 μl of Dynabeads magnetic beads at 4 °C under agitation. Samples were washed five times with 400 μl CHAPS buffer, re-suspended in 100 μl of 2X SDS Laemmli buffer and heated at 90 °C for 10 min before electrophoresis. No c-FLIP protein was immunoprecipitated after incubation with beads alone or with the control IgG antibody.

HER3/c-FLIP_L/S_ double immunoprecipitation was performed after NRG1 stimulation and/or 9F7-F11 incubation of BxPC3 cells (Fig. 3). First, total cell lysates (2 mg) were incubated with 2 μg of the anti-HER3 antibody 2F12, which recognizes the HER3 intracellular C-terminal tail and does not compete with 9F7-F11. The incubation was performed at 4 °C for 6 h before overnight incubation with 20 μl of magnetic Dynabeads at 4 °C under agitation. Total supernatants were recovered and then incubated with 2 μg of the anti-c-FLIP antibody H-202 at 4 °C for 6 h, before overnight incubation with 20 μl of Dynabeads magnetic beads at 4 °C under agitation. Samples were then processed as described above before electrophoresis.

DR5-DISC was isolated after immunoprecipitation (Fig. 6) using the Dynabeads system, as described by the manufacturer. Briefly, 2 mg of total cell lysates from 9F7-F11-treated BxPC3 cells were incubated with magnetic beads previously cross-linked with the anti-DR5 antibody D4E7. The Dynabeads bead-coupled DISC complexes were immunoprecipitated overnight at 4 °C in a rotator, and then washed ten times with CHAPS buffer, before electrophoresis and western blotting.

### Western blotting

2 × 10^6^ cells/dish were cultured at 37 °C for 24 h. After serum starvation in RPMI/1%serum with antibiotics for 24 h, cells were incubated with various compounds. For short kinetics, cells were stimulated with 100 ng/ml of NRG1 and/or incubated with 50 μg/ml of 9F7-F11 for 15 min to 3 h. For long kinetics, cells were incubated with 50 μg/ml of anti-HER3 antibody 9F7-F11 for 24 h to 120 h. After incubation, cells were washed, scraped and lysed with CHAPS buffer (Sigma-Aldrich), as indicated above. After washing in 1X PBS, the insoluble fraction was removed by centrifugation, and protein concentration in cell lysates was determined using the BCA assay. Two hundred micrograms of protein lysates were directly mixed with Laemmli buffer and heated at 95 °C for 5 min. After electrophoresis in reducing conditions, proteins were transferred to polyvinylidenedifluoride membranes (Millipore) and then incubated with the relevant primary and peroxidase-conjugated secondary antibodies, as previously described [33]. β-tubulin or β-actin were used as loading control.

### Statistical analysis

Apoptosis values represent the means ± SD for at least three independent experiments performed in triplicates. The significance of differences between experimental variables was determined using the two-tailed Student’s *t* test. The significance of *P* values are *P < 0.05, **P < 0.01 or ***P < 0.001.

## Results

### The anti-HER3 antibody 9F7-F11 induces tumor cell apoptosis through caspase-8/9/3 activation and PARP cleavage

**Fig. 1:**
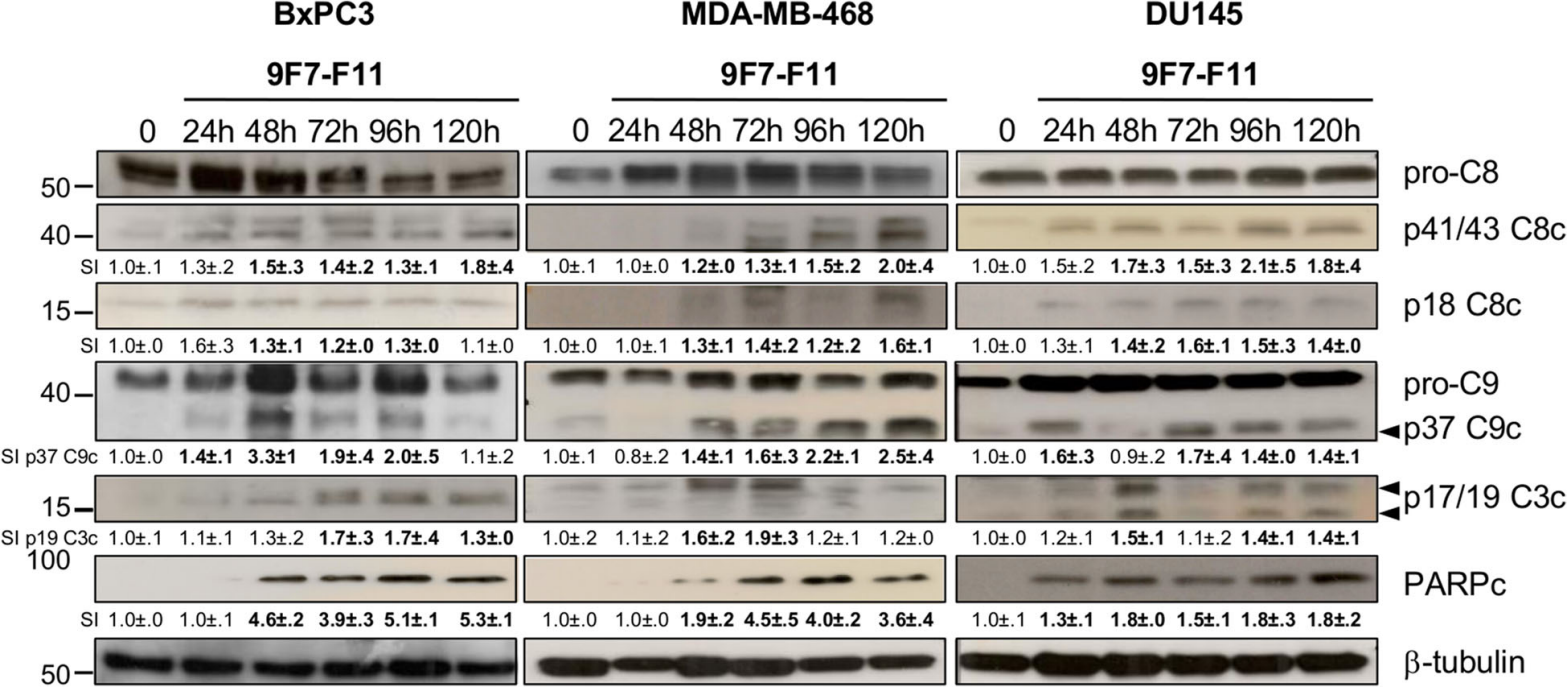
9F7-F11-induced tumor cell apoptosis occurs through caspase-8/9/3 activation and PARP cleavage. BxPC3, MDA-MB-468 or DU145 cancer cells were incubated with the anti-HER3 antibody 9F7-F11. Caspase-8, − 9 and − 3 and PARP cleavage were analyzed by western blotting and cell lysates prepared at different time points during antibody incubation. Quantification of signal intensity (SI) with ImageJ software is indicated below the images (relative to untreated control measured as 1.0 ± .0). Significant increase or decrease of the densitometry, compared to control, is indicated in bold. M: medium alone; p41/43 and p18 C8c: caspase-8 cleavage; p37 C9c: caspase-9 cleavage; p17/19 C3c: caspase-3 cleavage; PARPc: PARP cleavage. β-tubulin was evaluated as loading control

We previously demonstrated by flow cytometry analysis after Annexin V/7-AAD staining that the anti-HER3 antibodies 9F7-F11, 16D3-C1 and H4B-121 induce apoptosis of pancreatic cancer cells [2]. To precisely determine the underlying molecular mechanisms, we assessed apoptosis induction in three cancer cell lines at different time points during incubation with the allosteric NRG1 non-competing antibody 9F7-F11 [2, 32, 33]. 9F7-F11 induced caspase-8 cleavage into the p41/43 and p18 C8c fragments in BxPC3 (pancreas), DU145 (prostate) and MDA-MB-468 (breast) at 48 h (Fig. 1). The p37 C9c cleavage product, which indicates activation of caspase-9, appeared at 24 h for BxPC3 and DU145 cell lines, and 48 h for MDA-MB-468. p17/19 C3c cleavage products, which indicate activation of caspase-3, mainly occured 48 h-post 9F7-F11 treatment for MDA-MB-498 and DU145, and 72 h-post treatment for BxPC3 (Fig. 1). Caspase activation was followed by PARP cleavage from 24 h–48 h to the end of the experiment (120 h) (Fig. 1). Incubation with the HER3 ligand NRG1 alone did not induce apoptosis BxPC3 cells (Additional file 1: Figure S2). Conversely, 9F7-F11 could induce apoptosis of BxPC3 cells (caspase-8/9/3 cleavage/activation and PARP cleavage) also in the presence of NRG1 (Additional file 1: Figure S2). This demonstrated that 9F7-F11-induced apoptosis via caspase activation is a general mechanism of cancer cell death, independent of ligand stimulation.

### 9F7-F11 induces c-FLIP proteasomal degradation

**Fig. 2:**
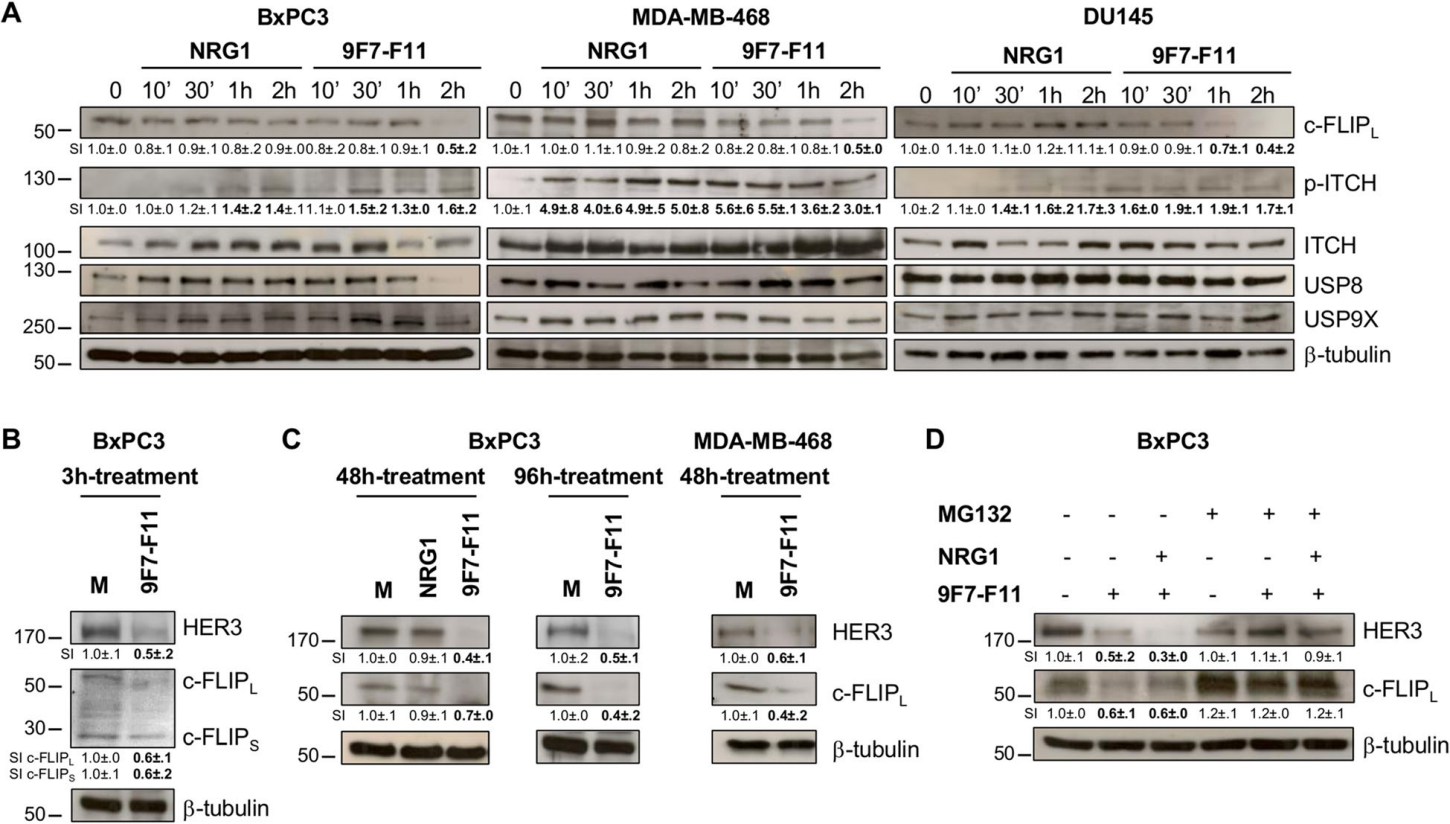
9F7-F11 induces c-FLIP downregulation by proteasomal degradation*.* **a** Cancer cells were incubated with NRG1 or 9F7-F11. After cell lysis at different time points, c-FLIP_L_, USP8 and USP9X expression, as well as ITCH expression and phosphorylation (p) were analyzed by western blotting. **b** BxPC3 cells were incubated with 9F7-F11 for 3 h. HER3, c-FLIP_L_ and c-FLIP_S_ expression were analyzed by western blotting. **c** BxPC3 and MDA-MB-468 cells were incubated with NRG1 or 9F7-F11 for 48 h or 96 h, before detection of HER3 and c-FLIP_L_ expression by western blotting. **d** After pre-incubation or not with 10 μM MG132 for 4 h, BxPC3 cells were incubated with 9F7-F11 with or without NRG1. After cell lysis, expression of HER3 and c-FLIP_L_ was assessed in whole cell lysates by western blotting. The rabbit anti-HER3 polyclonal antibody C-17 (Santa Cruz Biotechnology) was used for detection. Protein level was measured with the ImageJ software and indicated as signal intensity (SI), relative to untreated control (SI = 1.0 ± .0). Significant increase or decrease of the densitometry, compared to control, is indicated in bold. β-tubulin was evaluated as loading control

We previously showed that 9F7-F11 promotes HER3 ubiquitination and proteasomal degradation through JNK1/2-dependent ITCH activation [33]. As c-FLIP also is an ITCH target [26, 27], we asked whether this anti-apoptotic factor was ubiquitinated and degraded concomitantly with HER3. Expression of the long isoform c-FLIP_L_ was notably reduced in BxPC3, MDA-MB-468, and DU145 cells after 2 h-incubation with 9F7-F11, but not with NRG1 (Fig. 2a). ITCH phosphorylation was increased at 10-30 min until the end of the experiment (2 h) (Fig. 2a). Expression of USP8 and USP9X, which stabilize ITCH by preventing its auto-ubiquitination [27, 37], was globally stable during the entire experiment, except for BxPC3 cells at 2 h post-9F7-F11 treatment. Both long and short c-FLIP isoforms (c-FLIP_L_ and c-FLIP_S_) and HER3 were downregulated after 3 h of incubation with 9F7-F11 (Fig. 2b). c-FLIP_L_ expression, as well as HER3 expression, was reduced in BxPC3 and MDA-MB-468 cells after longer incubation with 9F7-F11, but not with medium or NRG1 (Fig. 2c). Then, to determine whether 9F7-F11 induced c-FLIP degradation via the proteasome, we pre-incubated, or not, cells with the proteasome inhibitor MG132 for 4 h, before addition of 9F7-F11 with or without NRG1 (Fig. 2d). Pre-incubation with MG132 inhibited the 9F7-F11-induced reduction in c-FLIP_L_ level, as previously reported for HER3 [33]. These findings demonstrated that 9F7-F11 reduces substantially c-FLIP and HER3 expression via proteasomal degradation, and independently of ligand stimulation.

### USP8-regulated ITCH binding to c-FLIP mediates c-FLIP ubiquitination in cancer cells incubated with 9F7-F11

**Fig. 3:**
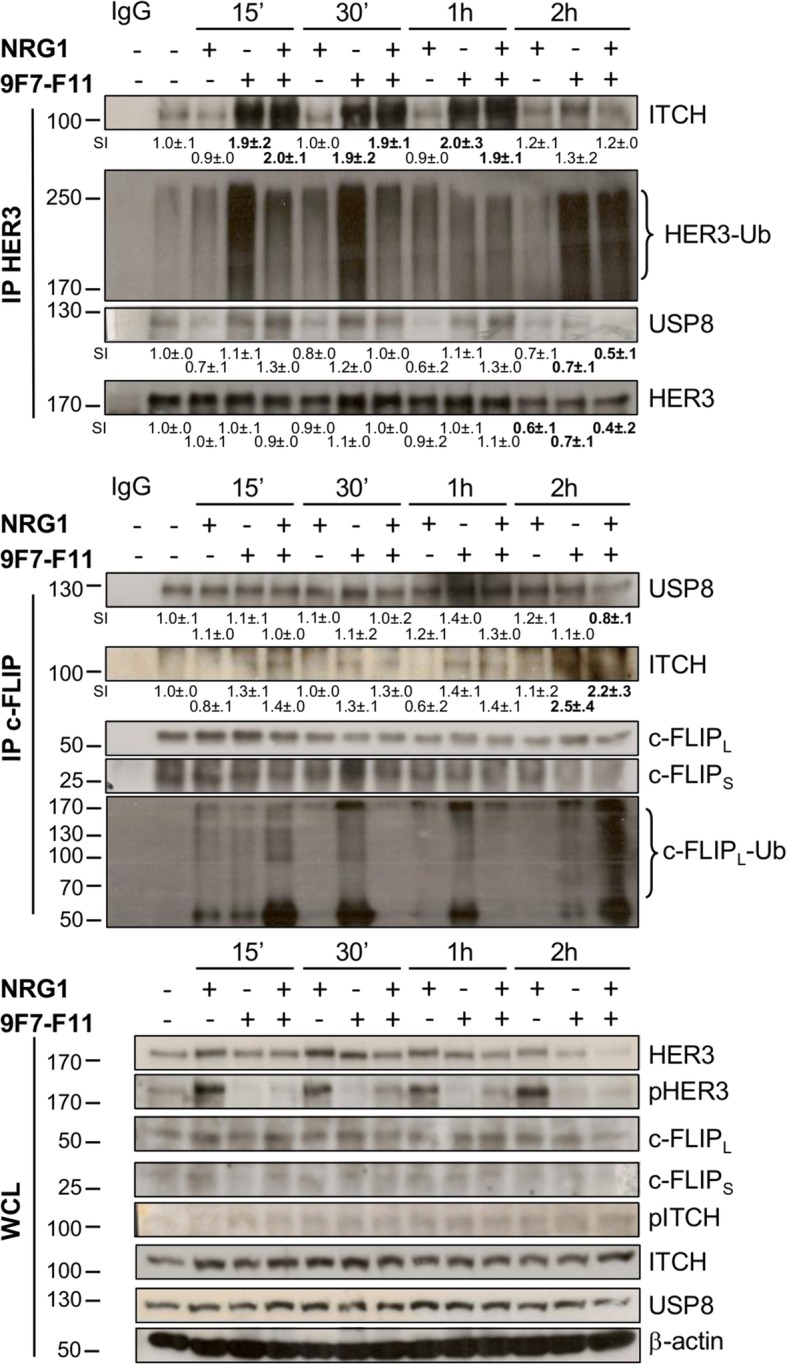
USP8-regulated ITCH interaction with c-FLIP mediates 9F7-F11-induced c-FLIP ubiquitination. BxPC3 cells were incubated with NRG1 or/and 9F7-F11 for various times. After cell lysis in CHAPS buffer, 2 mg of total protein extracts were co-immunoprecipitated with the anti-HER3 antibody 2F12 (Millipore) against HER3 C-terminal tail. Then, the first soluble supernatant was co-immunoprecipitated with the rabbit anti-c-FLIP polyclonal antibody H-202 (Santa Cruz Biotechnology) that targets both c-FLIP_L_ and c-FLIP_S_. The presence of ITCH and USP8 in the two immunoprecipitates was assessed by western blotting. HER3 and c-FLIP ubiquitination status were assessed using the anti-K48 ubiquitin antibody. Whole cell lysates (WCL) were analyzed using the appropriate antibodies. Quantification of signal intensity (SI) with ImageJ software is indicated below the images, in comparison to SI = 1.0 ± .0 for untreated control. Significant increase or decrease of the densitometry, compared to control, is indicated in bold. β-actin was evaluated as loading control

To determine whether c-FLIP binds to ITCH following antibody treatment, we collected BxPC3 cells at different time points during incubation with NRG1 or/and 9F7-F11, and immunoprecipitated them with anti-HER3 and anti-c-FLIP antibodies. We observed ITCH co-immunoprecipitation with HER3, and HER3 ubiquitination after 15 min of incubation with 9F7-F11 (with or without NRG1), and HER3 degradation at 2 h (Fig. 3, upper panel). In c-FLIP co-immunoprecipitation, strong c-FLIP_L_ ubiquitination at 2 h was correlated with stronger ITCH interaction (Fig. 3, middle panel). ITCH was not co-immunoprecipitated with c-FLIP_L_ in BxPC3 cells incubated with medium or NRG1 alone (Fig. 3, middle panel), suggesting that ITCH needs to be activated for substrate binding, as shown in HEK293 cells [38] and in our previous study [33].

USP8 interacts and stabilizes c-FLIP by deubiquitination, leading to DR-mediated apoptosis suppression [39]. USP8 also interacts with and stabilizes ITCH to induce c-FLIP ubiquitination, upon AKT inhibition, leading to TRAIL-induced apoptosis [27]. We already demonstrated that USP8 controls ITCH stability during 9F7-F11-induced HER3 ubiquitination and degradation [33]. Here, we found that USP8 was present in the c-FLIP_L_-ITCH complex in untreated cells, but was slightly reduced after 2 h of 9F7-F11 incubation (Fig. 3, IP c-FLIP), as previously reported [39]. USP8 was constitutively present also in the HER3-ITCH complex, and its expression slightly increased upon incubation with 9F7-F11 up to 1 h (Fig. 3, IP HER3), as we previously demonstrated [33]. The strong reduction of USP8 level in the two complexes at 2 h suggests that after contributing to the formation of these complexes, USP8 leaves them to favor c-FLIP_L_ and HER3 downregulation. Altogether, these results demonstrated that 9F7-F11 induced USP8 recruitment to stabilize ITCH, and then, the USP8-ITCH complex binds to the ITCH targets c-FLIP_L_ and HER3, allowing their ubiquitination and proteasomal degradation.

### ITCH and USP8 silencing by siRNA inhibits 9F7-F11-induced c-FLIP ubiquitination and proteasomal degradation

**Fig. 4:**
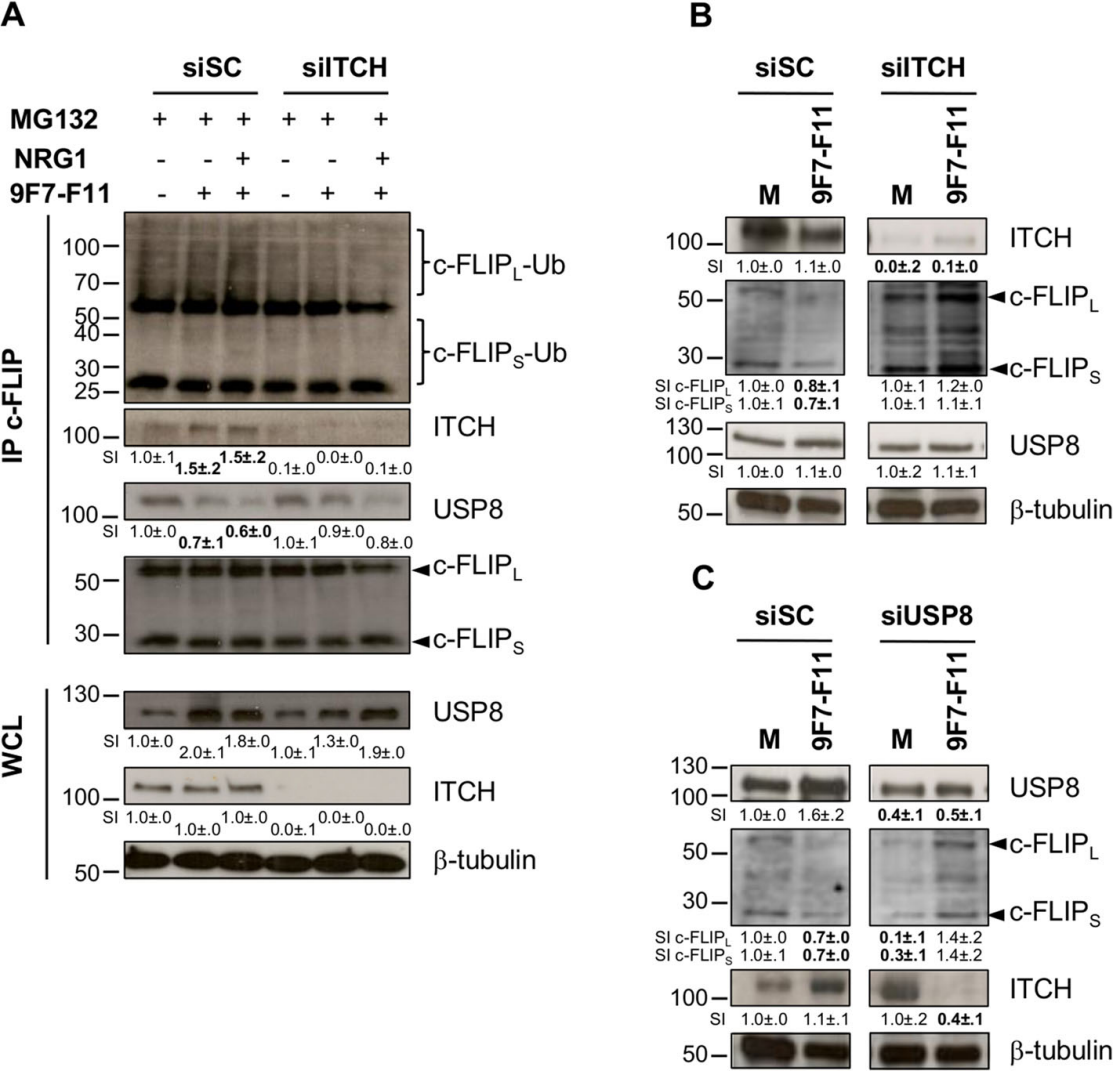
ITCH or USP8 silencing by siRNA inhibits 9F7-F11-induced c-FLIP ubiquitination and proteasomal degradation. **a** BxPC3 cells were transfected with 50 nM ITCH-specific siRNA (siITCH) or with control scramble siRNA (siSC) for 72 h, before pre-treatment with 10 μM MG132 for 4 h. Cells were then incubated with 9F7-F11, with or without NRG1, or medium as control for 4 h. After immunoprecipitation of total protein extracts (2 mg) with the anti-c-FLIP antibody H-202, c-FLIP, ITCH and USP8 expression and c-FLIP ubiquitination were analyzed by western blotting. BxPC3 cells were transfected with siSC*,* siITCH (**b**) or siUSP8 (**c**) for 72 h, and then incubated with 9F7-F11 for 4 h. Expression of ITCH, c-FLIP and USP8 was assessed in total protein extracts by western blotting. Protein level was measured with the ImageJ software and indicated as signal intensity (SI), relative to untreated control (SI = 1.0 ± .0). Significant increase or decrease of the densitometry, compared to control, is indicated in bold. β-tubulin was evaluated as loading control

To confirm ITCH involvement in antibody-induced c-FLIP ubiquitination, we analyzed c-FLIP ubiquitination after immunoprecipitation using an anti-c-FLIP antibody of protein extracts from BxPC3 cells transfected with anti-*ITCH* (siITCH) or scramble control (siSC) siRNAs and pre-treated with MG132 before incubation with 9F7-F11 and/or NRG1 for 4 h (Fig. 4a). We observed c-FLIP_L_ poly-ubiquitination and ITCH co-immunoprecipitation in siSC cells incubated with 9F7-F11 alone or with NRG1, but not in siITCH cells (Fig. 4a). Moreover, in siSC cells, 9F7-F11 reduced USP8 interaction with c-FLIP_L_ and promoted ITCH recruitment, allowing c-FLIP_L_ ubiquitination (Fig. 4a). Western blot analysis confirmed c-FLIP_L_ and c-FLIP_S_ degradation in siSC cells upon incubation with 9F7-F11 alone (Fig. 4b). This effect was abrogated by siRNA-mediated silencing of *ITCH* (Fig. 4b) and *USP8* (Fig. 4c). These results underlined ITCH and USP8 main role in 9F7-F11-induced c-FLIP degradation through ubiquitination. Finally, ITCH downregulation in 9F7-F11-treated siUSP8, but not in siSC cells (Fig. 4c) suggests that USP8 deubiquitinates and stabilizes ITCH, which then promotes rapid c-FLIP ubiquitination and proteosomal degradation [33]. In control cells (medium alone), USP8 knock-down induced c-FLIP downregulation, as compared with siSC treated cells (Fig. 4c), confirming that USP8 directly deubiquitinates c-FLIP [39].

### ITCH silencing or chemical inhibition blocks 9F7-F11-induced apoptosis and c-FLIP degradation

**Fig. 5:**
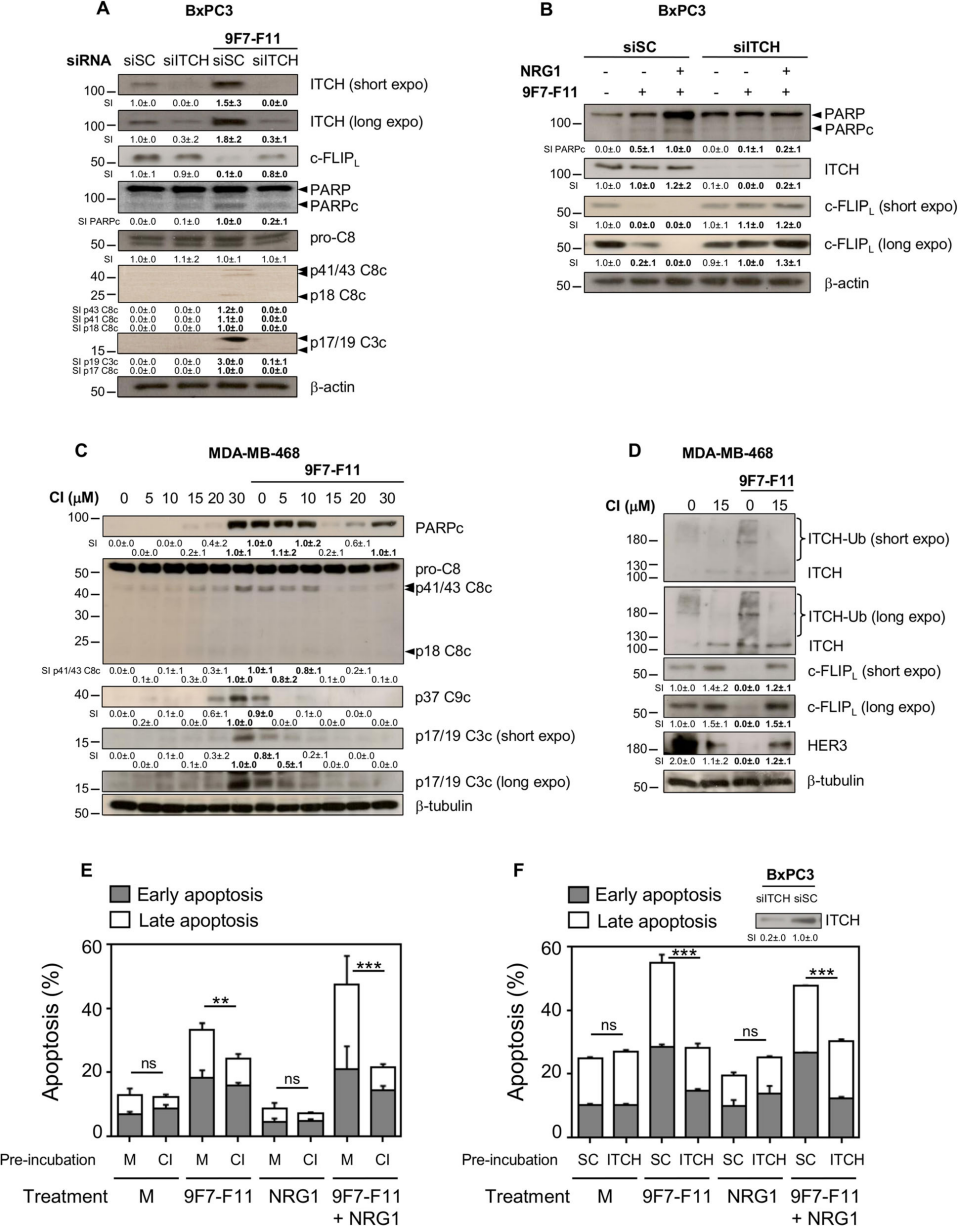
ITCH silencing or chemical inhibition blocks 9F7-F11-induced apoptosis and c-FLIP degradation. siSC- and siITCH-transfected BxPC3 cells were incubated with 9F7-F11 alone (**a**), or with NRG1 (**b**) for 48 h before detection by western blotting of ITCH and c-FLIP_L_ expression, and PARP/caspase cleavage. MDA-MB-468 cells were incubated with increasing doses of chlorimipramine (CI) for 24 h (**c**), or with 15 μM CI (**d**) before incubation with 9F7-F11 for 24 h. Total protein extracts were analyzed by western blotting to evaluate PARP and caspase cleavage (**c**), ITCH ubiquitination and expression and c-FLIP and HER3 expression (**d**). Quantification of signal intensity (SI) with ImageJ software is indicated below the images. No protein expression was measured as 0.0 ± .0. Significant decrease or increase of the densitometry, compared to control, is indicated in bold. BxPC3 cells were left untreated or pre-incubated with ITCH chemical inhibitor CI for 48 h before treatment with 9F7-F11 with or without NRG1. Apoptosis was measured at 96 h by flow cytometry after cell labelling with Annexin V/7-AAD (**e**). siSC and siITCH-transfected BxPC3 cells were treated with 9F7-F11 alone or with NRG1. Western blot was performed to check ITCH reduction in siITCH-transfected BxPC3 cells. Apoptosis was measured at 96 h by flow cytometry after cell labelling with Annexin V/7-AAD (**f**). **P < 0.01, ***P < 0.001, ns not significant

To determine whether ITCH-induced c-FLIP_L_ degradation had a major role in anti-HER3 antibody-induced cancer cell apoptosis, we assessed PARP and caspase-8/3 cleavage by western blotting in siITCH and siSC BxPC3 cells incubated with 9F7-F11 alone (Fig. 5a) or with NRG1 (Fig. 5b) for 48 h. In siITCH cells, ITCH expression was strongly downregulated, and 9F7-F11-induced c-FLIP_L_ degradation inhibited, compared with siSC cells (Fig. 5a). This effect was stronger in siITCH cells co-incubated with NRG1 and 9F7-F11 (Fig. 5b). Inhibition of c-FLIP_L_ degradation repressed 9F7-F11-induced apoptosis, as indicated by the lower level of cleaved PARP, p41/43 and p18 fragments (caspase-8 cleavage), and p17/19 fragments (caspase-3 cleavage) in siITCH cells compared with siSC cells (Fig. 5a). We confirmed the requirement of ITCH-mediated c-FLIP_L_ proteasomal degradation for 9F7-F11-induced apoptosis using chlorimipramine (Cl), a specific ITCH chemical inhibitor that is known to induce apoptosis at high dose, and irreversibly blocks ITCH by binding to its substrate pocket [40]. In the absence of 9F7-F11 antibody, incubation with high dose of CI (30 μM) induced apoptosis of MDA-MB-468 cells, as indicated by PARP and caspase-8/9/3 cleavage (Fig. 5c), as previously described [40, 41]. However, pre-incubation with 15 μM CI for 48 h completely repressed 9F7-F11-induced caspase activation and apoptosis (Fig. 5c), probably by inhibiting ITCH binding to its substrate. When we combined higher dose (30 μM) of CI with 9F7-F11, we restored PARP cleavage induced by CI, albeit antibody-induced apoptosis is still repressed. ITCH blockade by 15 μM CI in 9F7-F11-treated cells was associated with the disappearance of ITCH ubiquitination, and inhibition of antibody-induced c-FLIP_L_ and HER3 degradation, compared with cells incubated with medium or 9F7-F11 alone (Fig. 5d). In the agreement, ITCH chemical inhibitor CI reduced the percentage of apoptotic cells from 47.5 to 21.5% in NRG1/9F7-F11-treated BxPC3 cells (Fig. 5e), with a stronger effect on late apoptosis; this reduction being also observed in CI-pre-incubated BxPC3 cells and further treated with 9F7-F11 alone, in comparison with medium-pre-incubated antibody-treated cells (Fig. 5e). Similarly, *ITCH* RNA silencing of NRG1/9F7-F11-treated BxPC3 cells (siITCH) decreased apoptosis to 29.4%, with regard to 47.8%-apoptosis observed in siSC cells (Fig. 5f). This reduction of apoptosis was also observed in siITCH BxPC3 cells treated with 9F7-F11 alone, compared with siSC cells (Fig. 5f). As positive control, staurosporine induced 68.4%-cell apoptosis at 20 h-post-treatment (Additional file 1: Figure S3). Taken together, these results emphasized that the anti-HER3 antibody 9F7-F11 induces ITCH activation, leading to HER3 and c-FLIP degradation to allow caspase-mediated apoptosis.

### 9F7-F11 activates the extrinsic apoptotic pathway through FAS and DR5 upregulation, TRAIL expression and DcR2 downregulation

**Fig. 6:**
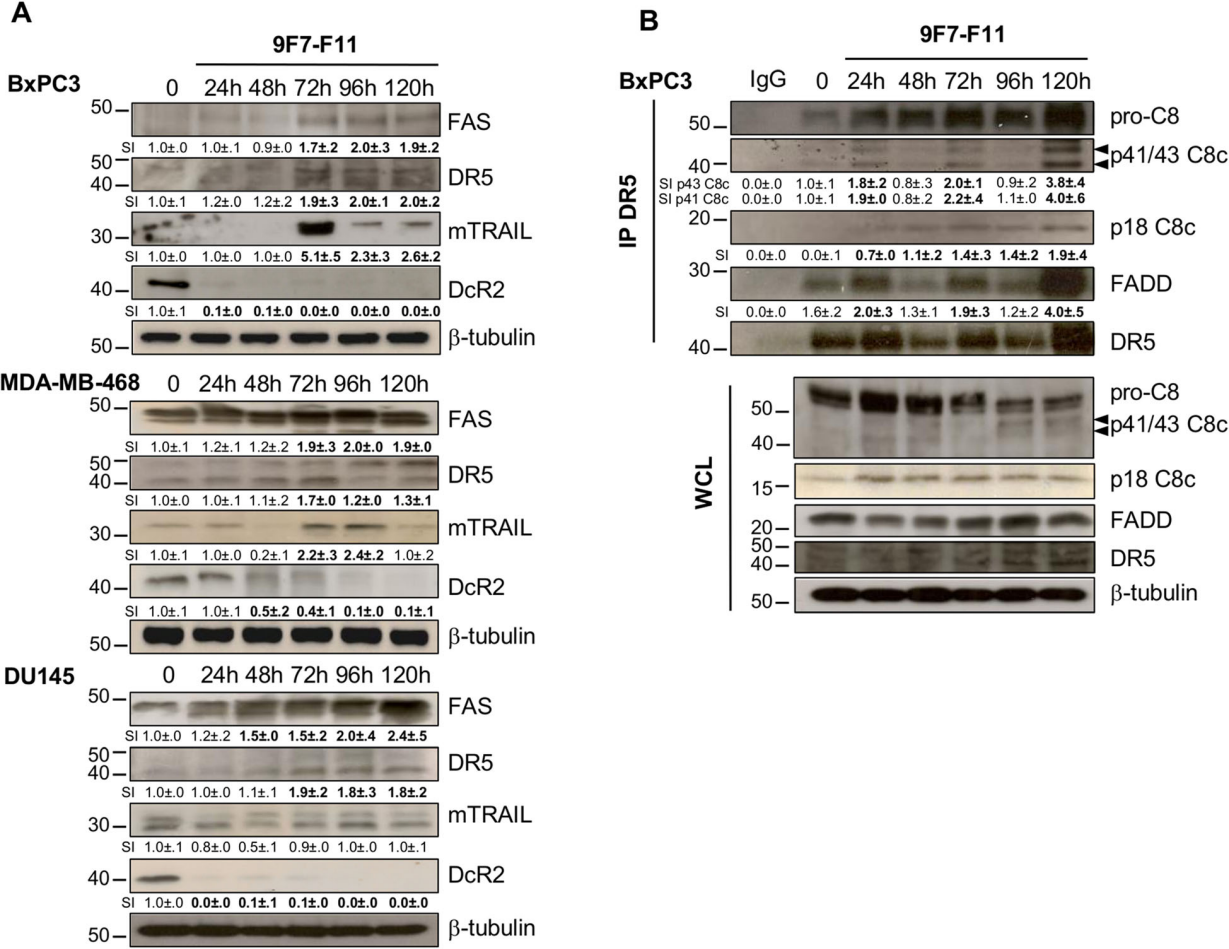
9F7-F11 activates the extrinsic apoptotic pathway through FAS and DR5 upregulation, TRAIL expression induction and DcR2 downregulation. **a** BxPC3, MDA-MB-468, and DU145 cells were treated with 9F7-F11 for various times. FAS, DR5, mTRAIL and DcR2 expression were detected by western blotting. **b** BxPC3 cells were incubated with 9F7-F11. After cells lysis at different time points, total protein extracts (2 mg) were co-immunoprecipitated with the human anti-DR5 polyclonal antibody D4E7 (Cell Signaling Technology) or with human anti-IgG as control. The presence of DISC components (caspase-8, FADD and DR5) was assessed in immunoprecipitates and whole cell lysates (WCL) by western blotting. Protein level was measured with the ImageJ software and indicated as signal intensity (SI), relative to untreated control (SI = 1.0 ± .0). Significant increase or decrease of the densitometry, compared to control, is indicated in bold. β-tubulin was evaluated as loading control

To test whether DRs were involved in 9F7-F11-induced apoptosis, we analyzed the expression of factors involved in the extrinsic pathway by western blotting after incubation of various cancer cell lines with 9F7-F11. Compared with untreated cells, the FAS and DR5 receptors were upregulated at 72 h of incubation until the end of the experiment (120 h) (Fig. 6a). TRAIL precursor (mTRAIL), which induces DR5 activation via a paracrine or autocrine loop, was also overexpressed at 72 h, except for DU145. The decoy receptor DcR2, which inhibits DR5 activation by trapping TRAIL, was already downregulated at 24-48 h (Fig. 6a). This demonstrated that 9F7-F11 induces the caspase-8-mediated extrinsic apoptotic pathway by downregulating DcR2, leading to DR5 upregulation and TRAIL expression.

To test whether activation of the extrinsic apoptotic pathway induces DISC formation, we immunoprecipitated BxPC3 cells with an anti-DR5 antibody at different time points during incubation with 9F7-F11 (Fig. 6b). In untreated cells (i.e., without caspase-8 activation to form a pre-DISC), FADD and pro-caspase 8 were weakly immunoprecipitated with DR5 compared to IgG baseline. Conversely, 9F7-F11 promoted p41/43 and p18 (caspase-8 cleavage) and FADD recruitment, indicating DR5-DISC formation. Interestingly, FADD activation, as well as p41/43 caspase 8 cleavage, seem to fluctuate with lower levels observed at 48 h and 96 h, and overexpression at 24 h, 72 h and 120 h-post 9F7-F11 treatment. Strong global activation was observed at 120 h-end of the experiment (Fig. 6b), thus correlating DR5/FADD overexpression with increased caspase-8 activation to induce the extrinsic apoptotic pathway.

### 9F7-F11 activates also the intrinsic mitochondrial apoptotic pathway

**Fig. 7:**
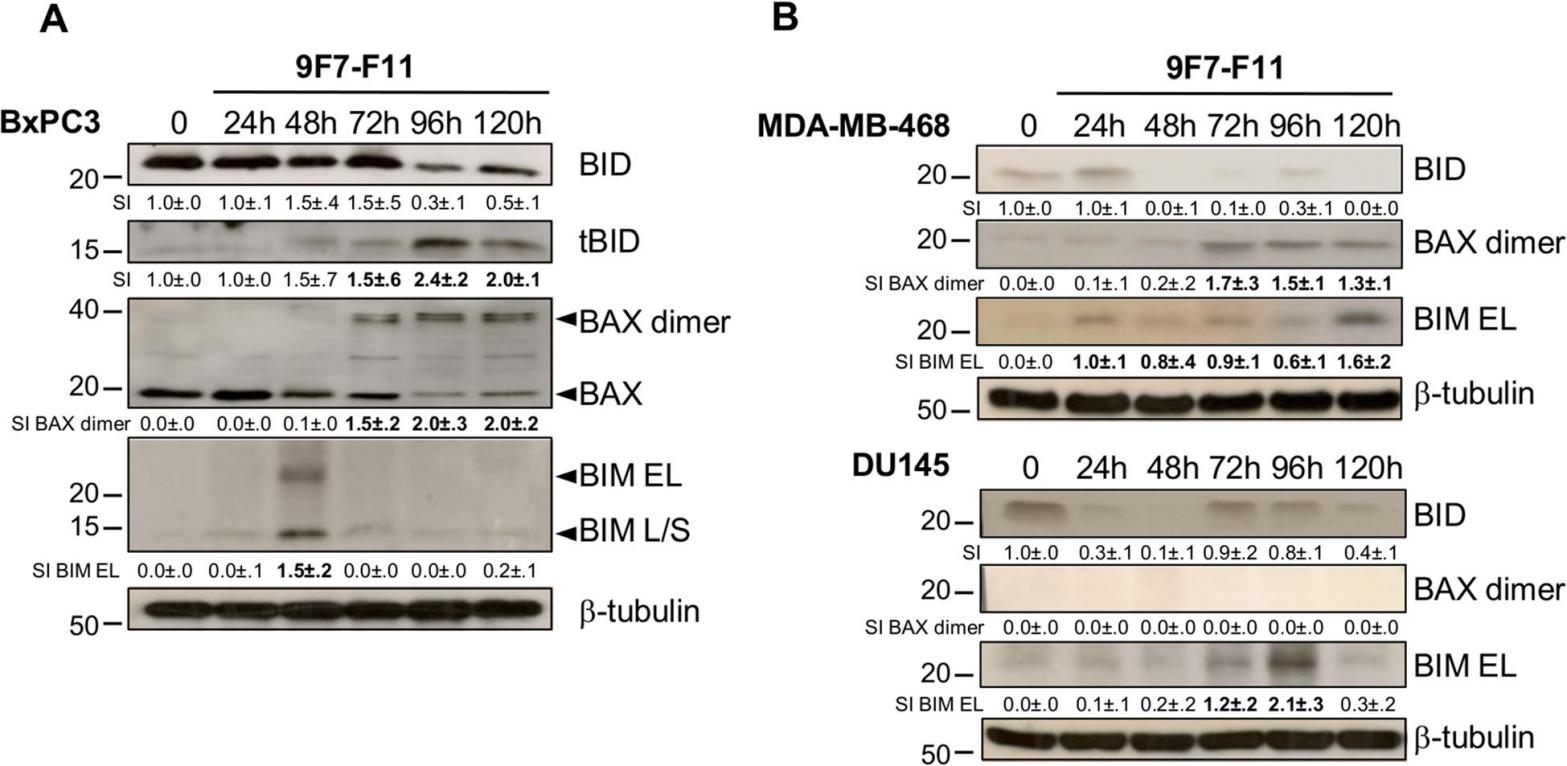
9F7-F11 activates the intrinsic mitochondrial apoptosis pathway by inducing caspase-8-mediated BID cleavage, BAX activation and BIM expression. BxPC3 (**a**), MDA-MB-468 and DU145 (**b**) cells were incubated with 9F7-F11 for various times. BID cleavage (tBID), BAX activation and BIM expression were analyzed by western blotting. Quantification of signal intensity (SI) with ImageJ software is indicated below the images. No protein expression was measured as 0.0 ± .0. Significant decrease or increase of the densitometry, compared to untreated control, is indicated in bold. β-tubulin was evaluated as loading control

9F7-F11 induced caspase-8 activation followed by caspase-9/3 cleavage (Fig. 1). Analysis by western blotting of caspase-mediated apoptosis in BxPC3 cells incubated with 9F7-F11 showed that the anti-HER3 antibody also induced BID cleavage and formation of the truncated form tBID from 72 h–96 h until the end of the experiment (120 h) (Fig. 7a), suggesting that caspase-8 activation induces BID cleavage to favor the mitochondrial death pathway. 9F7-F11 also upregulated BAX and promoted dimer formation at 72 h (Fig. 7a), strengthening the involvement of mitochondria to promote complete activation of caspase-3 via caspase-9. The induction of the p53 transcriptional target BIM (at 48 h; Fig. 7a) allows mitochondria amplification of caspase-3 activation. These effects were confirmed also in MDA-MB-468 cells incubated with 9F7-F11 in a slight different time-frame (Fig. 7b). We only observed 9F7-F11-induced BIM induction in BAX-deficient DU145 cells [42] (Fig. 7b). These results demonstrated the involvement of the intrinsic apoptotic pathway (BID truncation, BAX upregulation and BIM expression to induce full caspase-9 activation) in cancer cells incubated with 9F7-F11.

### 9F7-F11 induces downregulation of the pro-survival proteins c-IAP2 and XIAP

**Fig. 8:**
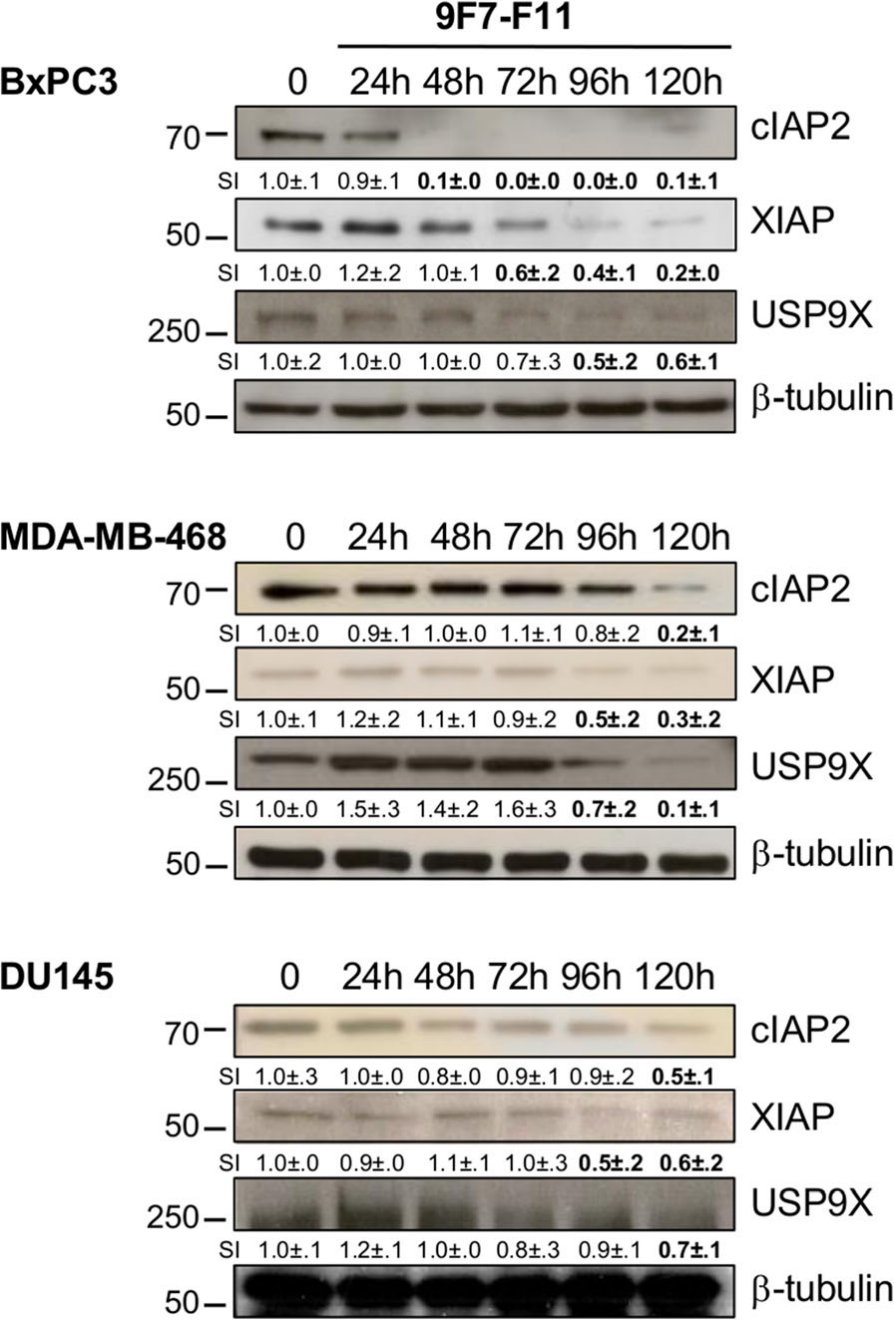
9F7-F11 induces downregulation of the pro-survival proteins c-IAP2 and XIAP. Cells were incubated with 9F7-F11, before western blot analysis of cIAP2 and XIAP (survival factors) and of UP9X (deubiquitinase) expression in protein lysates prepared at different time points. Quantification of signal intensity (SI) with ImageJ software is indicated below the images (relative to untreated control measured as 1.0 ± .0). Significant increase or decrease of the densitometry, compared to control, is indicated in bold. β-tubulin was evaluated as loading control

9F7-F11 sensitized tumor cells to caspase-mediated apoptosis through ITCH-dependent c-FLIP downregulation. Pro-survival proteins from the IAP family inhibit apoptosis [43]. For efficient apoptosis inhibition, XIAP is stabilized by interaction with the deubiquitinase USP9X and then promotes caspase-3 degradation through ubiquitination [44]. We previously demonstrated that anti-HER3 antibodies inhibit XIAP phosphorylation to favor apoptosis [2]. Here, western blotting analysis (Fig. 8) showed that in 9F7-F11-treated BxPC3 cells, cIAP2 and XIAP expression was reduced starting at 48 h–72 h and completely downregulated at 96 h. The progressive USP9X downregulation, which occurred concomitantly with that of cIAP2 and XIAP, could be responsible for XIAP degradation. We obtained similar results with other cancer cell lines (MDA-MB-468 and DU145 cells), but with slower kinetics (Fig. 8). This indicated that 9F7-F11 induces USP9X downregulation leading to XIAP degradation, which allows DR5-mediated caspase-dependent apoptosis.

## Discussion

**Fig. 9:**
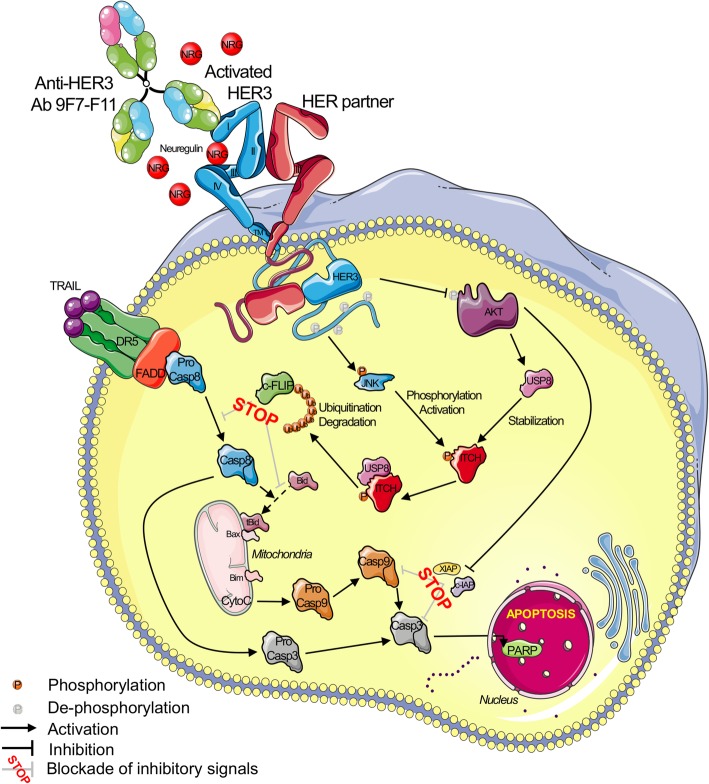
The representation illustrates a proposed model for ITCH-dependent proteasomal degradation of c-FLIP induced by 9F7-F11 to promote DR5/caspase-8 apoptosis. See text for details

Here, we report that ITCH-dependent proteasomal degradation of c-FLIP induced by the anti-HER3 antibody 9F7-F11 favors DR5/caspase 8-mediated apoptosis of tumor cells. This mechanism (as illustrated in Fig. 9) can explain how anti-HER3 antibodies directly induce cancer cell apoptosis [2, 45, 46] and counteract the HER3-mediated apoptosis inhibition observed in cancer cells resistant to chemotherapy [3, 4]. HER3 silencing [1, 47] or pharmacological inhibition [2] directly restores tumor-specific apoptosis, underlying its critical role in cell death inhibition. HER3 degradation induced by specific antibodies [2, 33, 48, 49] is a marker of pre-clinical drug efficacy [2, 32, 45, 46], and is frequently associated with cancer cell apoptosis induction [2, 45, 46]. We previously demonstrated that in cancer cells, 9F7-F11 blocks the PI3K/AKT pathway [2, 32, 33], induces HER3 downregulation and promotes cell apoptosis [2, 33], leading to in vivo tumor regression [2, 32]. The binding to HER3 and biological effects on tumor cells of 9F7-F11 are paradoxically facilitated by the natural ligand NRG1 [32]. By hijacking NRG1 addiction of cancer cells to promote its inhibitory effects on NRG1-mediated tumor growth and resistance, the allosteric non NRG1-competing 9F7-F11 displays a unique potential for targeted treatment of NRG1-positive cancers [32].

HER3 ubiquitination and degradation induced by 9F7-F11 mainly occur through JNK1/2-dependent ITCH activation, and are regulated by the deubiquitinases USP8 and USP9X [33]. Here, we showed that upon incubation of cancer cells with 9F7-F11, the E3 ubiquitin ligase ITCH interacts with c-FLIP to trigger c-FLIP ubiquitination and degradation, concomitantly with early ITCH recruitment to HER3 [33]. In this setting, 9F7-F11 induces JNK1/2 activation to phosphorylate ITCH on Thr222. Other studies identified JNK1/2 as the main regulator of ITCH-induced c-FLIP degradation after TNFα stimulation [26] or AKT inhibition in glioblastoma [27]. c-FLIP degradation via JNK/ITCH activation has been recently described to sensitize tamoxifen-resistant breast cancer to TRAIL-induced cell death [28]. 9F7-F11 could be useful to bypass resistance to chemotherapy in breast cancer by favoring c-FLIP degradation via JNK/ITCH activation. In basal conditions (medium alone), the deubiquitinase USP8 contributes to stabilization of c-FLIP, as shown here (Figs. 3 and 4) and by others [39], and of ITCH [27, 33]. Conversely, during incubation with 9F7-F11, USP8 leaves the ITCH-c-FLIP complex, allowing JNK1/2-mediated ITCH activation for c-FLIP ubiquitination. ITCH and USP8 silencing experiments highlighted the role of this ubiquitination/deubiquitination process in modulating 9F7-F11-induced c-FLIP and HER3 degradation, and also in the inhibition of caspase-8-mediated apoptosis of tumor cells. This links c-FLIP downregulation by 9F7-F11 with antibody-induced caspase-8 activation. In addition, antibody treatment disrupted the basal USP8-HER3 interaction to favor ITCH-mediated HER3 ubiquitination and proteasomal degradation. In conclusion, the deubiquitinase USP8 acts by co-regulating c-FLIP (Fig. 4), ITCH and HER3 [33] stability, and this triple regulation is affected by 9F7-F11. Interestingly, it has been reported that a synthetic USP8 inhibitor also induces downregulation of receptor tyrosine kinases, including HER3 and c-MET, leading to inhibition of cell survival/proliferation and tumor regression in mice xenografted with gefitinib-resistant non-small cell lung cancer cells [50]. Similarly to 9F7-F11 activity, vitamin E analogues (α-tocopherol derivatives) inhibit the HER3-mediated AKT pro-survival pathway and the anti-apoptosis factors c-FLIP and survivin to favor caspase-mediated apoptosis in cisplatin-resistant ovarian cancer [51]. Targeting c-FLIP has been also proposed in various cancers, mainly indirectly by using chemotherapies such as cisplatin, 5-fluorouracil, gemcitabine, etoposide and paclitaxel [14].

We demonstrated that 9F7-F11 induces DR5 upregulation and TRAIL expression, leading to DR5-mediated caspase-8 activation through the formation of the DR5-DISC complex. Chemical compounds, such as dibenzylideneacetone, also upregulate DR5, via activation of ROS-mediated C/EBP homologous transcription factor (CHOP) that induces transcription of pro-apoptotic proteins [52]. CHOP-dependent DR5 upregulation and ROS production promote TRAIL-induced apoptosis through downregulation of XIAP, survivin, and c-FLIP_L_ and c-FLIP_S_ [52]. Similarly, the natural molecule zerumbone, a sesquiterpene from tropical ginger, induces DR5 upregulation via ROS-mediated activation of the MAP kinases ERK1/2 and p38, leading to DR5/TRAIL-mediated apoptosis [53]. Oxidative stress, through ROS production, is often associated with apoptosis via JNK1/2 activation. Some therapeutic antibodies promote apoptosis together with ROS accumulation, leading to JNK activation [54, 55]. Anti-HER3 antibodies, such as MM-121 and hMP-RM-1 that downregulate survivin [4], or 9F7-F11 that inhibits XIAP phosphorylation [2], favor apoptosis and reduce cell survival and proliferation. Here, we showed that 9F7-F11 represses the expression of the survival proteins XIAP and c-IAP2 and also of c-FLIP, concomitantly with DR5 upregulation and TRAIL expression induction. This suggests that antibody-induced caspase-8-mediated apoptosis involves DR5 activation by TRAIL autocrine loop or by ROS-dependent DR5 aggregation for DISC formation. It is worth noting that in our setting, DR5-DISC formation started early with the recruitment of FADD and pro-caspase 8, before DR5 upregulation. Therefore, anti-HER3 antibody-triggered DR5 aggregation and DISC formation might occur independently of TRAIL induction to activate caspase-8-mediated apoptosis. Our findings showed that 9F7-F11-induced apoptosis involves also the mitochondrial pathway with the induction of BID cleavage by caspase-8, BAX upregulation and BIM expression induction, leading to mitochondria-dependent caspase-9 activation and full caspase-3 activation. This is in agreement with previous work showing that siRNA-mediated HER3 downregulation induces BAX-BAK-mediated apoptosis [47]. Upon incubation with 9F7-F11, we observed BID cleavage and BIM expression also in DU145 prostate cancer cells, which are BAX-deficient [42] but express BAK that could replace BAX to induce caspase-9 activation. It has been demonstrated that BAK is preferentially activated by tBID and BAX by BIM, to modulate the response to chemotherapy [56]. Indeed, anti-HER3 antibody-induced apoptosis in DU145 cells was lower than in the other tested cancer cell lines, probably because BAX deficiency prevented BIM-mediated apoptosis.

## Conclusions

We provide evidence that the allosteric non-NRG1 competing modulator 9F7-F11, sensitizes tumor cells to caspase-mediated apoptosis through ITCH-dependent degradation of c-FLIP, and independently of ligand addiction. The description of the multiple modes of action of the anti-HER3 antibody 9F7-F11 not only adds to our basic understanding of dysregulated signaling in cancer, but might help the selection of drug combinations and clinical indications for 9F7-F11 in NRG1-addicted or NRG1-rearranged cancer.

## Additional file

Additional file 1: **Figure S1.** (**a**) Flow cytometry analysis of EGFR, HER2 and HER3 expression at the membrane of BxPC3, DU-145 and MDA-MB-468 cells. (**b**) Western blot analysis of receptor and NRG1 expression, and USP8, USP9 and ITCH expression in whole lysates of BxPC3, DU-145 and MDA-MB-468 cell lines. (PDF 230 kb)

## Data Availability

All data generated or analysed during this study are included in this published article (and its supplementary information files).

## Abbreviations

7-AAD: A-Aminoactinomycin D
BAD: Bcl-2-associated death promoter
BID: BH3 interacting-domain death agonist
BIM: Bcl-2-like protein 11
c-FLIP: FLICE-like inhibitory protein
CI: Chlorimipramine
cIAP2: Cellular inhibitor of apoptosis protein 2
DED: Death effector domain
DISC: Death-inducing signaling complex
DR: Death receptor
FADD: Fas-associated protein with death domain
ITCH: E3 ubiquitin-protein ligase Itchy homolog
JNK: c-Jun N-terminal kinase
NRG1: Neuregulin 1
PARP: Poly (ADP-ribose) polymerase
SD: Standard deviation
USP8: Ubiquitin-specific peptidase 8
XIAP: X-linked inhibitor of apoptosis protein
