# Supplementary material for: ITCH-dependent proteasomal degradation of c-FLIP induced by the anti-HER3 antibody 9F7-F11 promotes DR5/caspase 8-mediated apoptosis of tumor cells
Source: Cell Commun Signal. 2019 Aug 23;17:106. doi: 10.1186/s12964-019-0413-8 (PMC6708219; doi:10.1186/s12964-019-0413-8)
Supplement: Supplementary file 1 — Figure S1. (a) Flow cytometry analysis of EGFR, HER2 and HER3 expression at the membrane of BxPC3, DU-145 and MDA-MB-468 cells. (b) Western blot analysis of receptor and NRG1 expression, and USP8, USP9 and ITCH expression in whole lysates of BxPC3, DU-145 and MDA-MB-468 cell lines. (PDF 230 kb) [file 12964_2019_413_MOESM1_ESM.pdf]

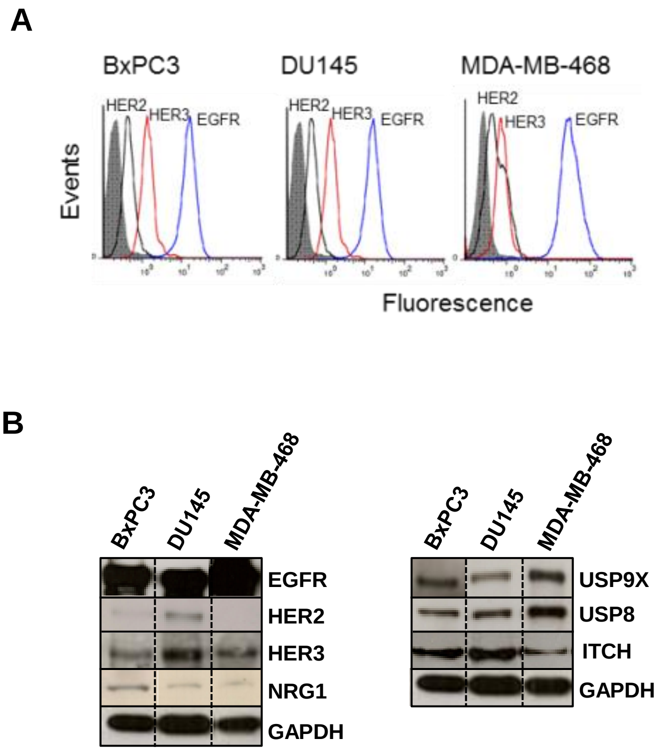

Figure S1. (**a**) Flow cytometry analysis of EGFR, HER2 and HER3 expression at the membrane of BxPC3, DU145 and MDA-MB-468 cells. (**b**) Western blot analysis of receptor and NRG1 expression, and USP8, USP9 and ITCH expression in whole cell lysates of BxPC3, DU145 and MDA-MB-468 cell lines.

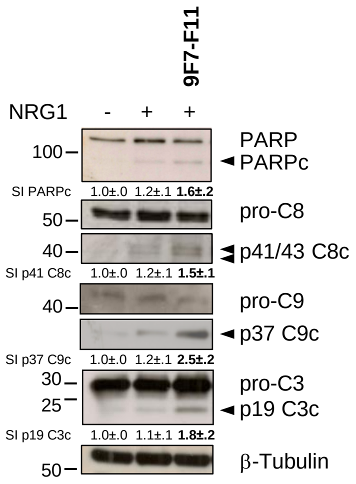

Figure S2. 9F7-F11-induced apoptosis of tumor cells occurs through activation of caspase-8/9/3 and PARP cleavage. BxPC3 cells were incubated with the anti-HER3 antibody 9F7-F11, and/or NRG1 for 60h. Total proteins extracts were analyzed by western blotting for caspase-8, -9 and -3, and PARP cleavage. Quantification of signal intensity (SI) with ImageJ software is indicated below the images (relative to untreated control measured as 1.0±.0). Significant increase or decrease of the densitometry, compared to control, is indicated in bold.

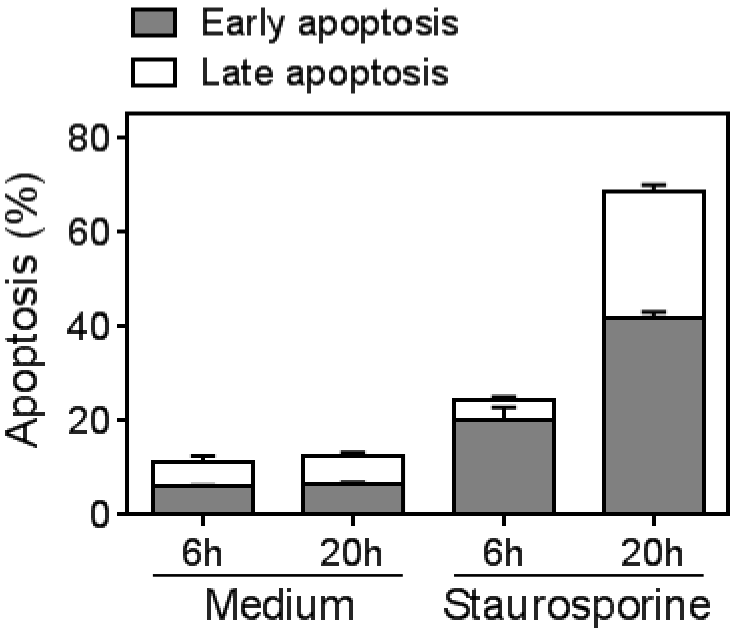

Figure S3. BxPC3 cells were treated with 300nM Staurosporine, as apoptosis positive control. Apoptosis was measured at 6h and 20h by flow cytometry after cell labelling with Annexin V/7-AAD.
